# Friction Stir Spot Welding of Aluminum and Copper: A Review

**DOI:** 10.3390/ma13010156

**Published:** 2019-12-31

**Authors:** Mingshen Li, Chaoqun Zhang, Dayong Wang, Li Zhou, Daniel Wellmann, Yingtao Tian

**Affiliations:** 1 Shanghai Key Laboratory of Digital Manufacture for Thin-Walled Structures, School of Mechanical Engineering, Shanghai Jiao Tong University, Shanghai 200240, China; 2 School of aerospace engineering, Shenyang Aerospace University, Shenyang 110136, China; 3 State Key Laboratory of Long-Life High Temperature Materials, Dong Fang Turbine Co., Ltd. Deyang Sichuan 61800, China; 4 Dongfang Electric Corporation Dongfang Turbine Co., LTD, Deyang 618000, China; 5 State Key Laboratory of Advanced Welding and Joining, Harbin Institute of Technology, Harbin 150001, China; 6 Shandong Provincial Key Laboratory of Special Welding Technology, Harbin Institute of Technology at Weihai, Weihai 264209, China; 7 Department of Engineering, Lancaster University, Bailrigg, Lancaster LA1 4YW, UK

**Keywords:** friction stir spot welding, aluminum, copper, dissimilar materials, intermetallic compounds

## Abstract

Aluminum (Al) and copper (Cu) have been widely used in many industrial fields thanks to their good plasticity, high thermal conductivity and excellent electrical conductivity. An effective joining of dissimilar Al and Cu materials can make full use of the special characteristics of these two metals. Friction stir spot welding (FSSW), as an efficient solid-state welding method suitable for joining of dissimilar metal materials, has great prospects in future industrial applications. In this paper, the FSSW studies on Al-Cu dissimilar materials are reviewed. The research progress and current status of Al-Cu FSSW are reviewed with respect to tool features, macroscopic characteristics of welded joints, microstructures, defects in welds and mechanical properties of joints. In addition, some suggestions on further study are put forward in order to promote the development and progress of Al-Cu FSSW studies in several respects: material flow, thermal history, addition of intermediate layer, auxiliary methods and functionalization of Al-Cu FSSW joint.

## 1. Introduction

At present, some structures need to have a variety of characteristics, and good and stable features to adapt to different service requirements. Therefore, in mechanical and electronic structures, connection of dissimilar materials is indispensable. The demand for these dissimilar joints has led to the rapid development of dissimilar materials joining technology [1,2]. Aluminum (Al) alloy is an ideal lightweight structural material with low density, high specific strength, good plasticity and other features [3]. Copper (Cu) material has high thermal conductivity, good corrosion resistance and excellent electrical conductivity [4,5]. In the electrical and refrigeration industries, the extensive application of Al and Cu materials makes the connection of these two materials inevitable [6,7]. Thus, realizing the sound joining of dissimilar Al-Cu materials has become a hot research topic, which is of great significance in promoting the development of the industry.

In the traditional fabrication processes of Al-Cu lap joints, mechanical joining and fusion welding are the commonly used methods. In mechanical joining, bolt joining [8] and rivet joining [9] increase the weight of the structure by introducing high-strength bolts and rivets, and the pre-drilled holes in them will cause stress concentration and affect the fatigue performance of the structure. Moreover, the reliable electrical conductivity of Al-Cu joints can hardly be achieved by mechanical joining method [10]. In fusion welding techniques, due to the different thermal physical properties of Al and Cu and the forming of hard and brittle intermetallic compounds (IMCs; abbreviations of technical terms presented in this work are collected in Table 1) at the interface, it is difficult to obtain Al-Cu joints with good metallurgical bonding and high strength [11,12]. Solid-state welding processes (FSW, FSSW, USW, EMPW, etc.) have been widely applied to retard the growth of brittle IMCs on the interfaces of dissimilar metals [13,14,15,16,17,18,19,20,21,22,23,24,25,26,27,28,29,30,31,32]. As a solid-state welding process, friction stir welding (FSW, invented by TWI, 1991 [13,14,15,16]) can avoid the aforementioned problems in Al-Cu fusion welding joints to a considerable degree due to its low welding temperature and the fierce stirring effect during dynamic welding process.

**Figure 1:**
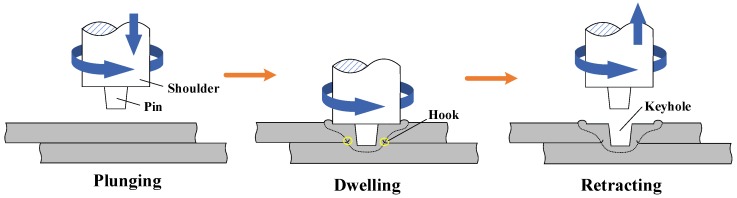
Schematic illustration of FSSW process.

In the manufacturing industry, some automation manufacturers, such as automobile and electronic equipment manufacturing, are more concerned with welding efficiency and energy saving; they typically choose to replace some dispensable seam welding processes with spot welding methods. Friction stir spot welding (FSSW) is a variant of FSW, which was proposed by Mazda Motor Company of Japan and then applied in production [33,34]. The FSSW process can be divided into three stages (Figure 1). In the first stage, the tool starts to rotate and plunge towards the plates. In the second stage, the rotating tool reaches the lowest point and maintains a dwelling time. At the last stage, the rotating tool withdraws and then the FSSW process completes. Different from the FSW process, the rotating tool in the FSSW process keeps no tilt angle or transverse movement. Therefore, the temperature distribution and material flow behavior in the FSSW process are quite different from those in the FSW process, and need to be studied separately. Up until now, the research on Al-Cu FSSW has been limited. These studies have mainly focused on the influence of the tool features [35,36], the optimization of welding parameters (rotational speed, plunge depth and dwelling time) [37,38,39,40,41], the welding thermal history [10,42], the IMCs in the joint interface [43,44] and their evolution [10,11].

Al-Cu dissimilar materials FSSW has great application prospects in automatic production line due to its effective and efficient characteristics of high joint strength and productivity. Nowadays, the researches of the Al-Cu FSSW need to develop in the direction of specification and systematization. In this paper, the tool features, macroscopic characteristics, microstructures, defects in welds, thermal behavior during welding and mechanical properties of Al-Cu FSSW joints are reviewed. Meanwhile, based on the relevant research results, interface bonding mechanisms and interfacial microstructure evolutions are analyzed and discussed.

## 2. Tool Features

The welding tool is the working part of FSSW equipment, and consists of a shoulder and a pin. During the FSSW process, the welding tool affects the heat production and material flow, which influences the microstructure and mechanical properties of the joint. Proper design of the welding tool is conducive to improving FSSW efficiency, increasing the window of FSSW process parameters and improving the quality of the Al-Cu FSSW joint.

In general, the shoulder of the rotating tool plays three roles in the FSSW welding process. First, the frictional heat between the shoulder and the workpiece provides most of the heat required for welding. Second, the shoulder drives the flow of upper plastic material with its rotation. Third, the shoulder forms a closed space with the un-plasticized material around the weld to prevent the upper part material from overflowing out of the weld. While the pin of the tool has less contribution to the FSSW heat input, and its main function is to drive the vertical flow of materials in the weld, especially when it is welding thick test plates. The combined effect of geometry and size of shoulder and pin determine the distribution of temperature field and the flow form of plastic material in the FSSW process. Therefore, the geometric design of the welding tool is necessary for obtaining high-quality Al-Cu FSSW joints. Several studies on the design and function of Al-Cu FSSW tool have been carried out. The welding tool of Al-Cu FSSW involved in the published papers are listed in Table 2.

**Figure 2:**
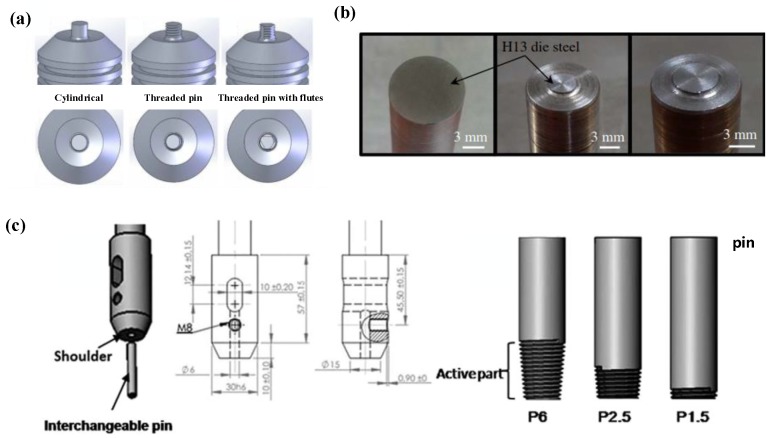
Welding tools with features of (**a**) featureless pin, threaded pin and threaded pin with flutes [42], (**b**) flat shoulder and short pin [36], and (**c**) flat shoulder and interchangeable threaded pin [45].

The geometry of the welding tool has a direct effect on the FSSW joint. The friction heat production and material flow of different shape welding tools are quite different, and are reflected in the mechanical properties of the joints. According to the research of Zhou et al. [42], among the three welding tools shown in Figure 2a (featureless pin, threaded pin and threaded pin with flutes), although the three had no obvious influence on the microhardness of the joint, the welding tool with the threaded pin possessed the highest failure load of Al-Cu FSSW joint, which was 4.3 kN, followed by the threaded pin with flutes and the featureless pin, with corresponding failure loads of 3.1 and 2.7 kN, respectively.

Moreover, Mubiayi et al. [35] studied the influence of the geometry of the welding tool on the Al-Cu FSSW joint by comparing a flat pin and flat shoulder (FPS) and a conical pin and concave shoulder (CCS); they reported that FPS mode possessed the highest shear load at 800 r/min, 1 mm shoulder plunge depth, while CCS mode possessed the lowest under the same parameters. In addition, with the rotation of the tool, Al particles were pressed into the vicinity of the Cu sheet. All joints produced by the CCS tool have a lower microhardness value near the region at the bottom of the lock hole, which is close to the average value of the Cu base material.

In addition to the geometry, the size of the welding tool is also a main factor affecting the strength of the Al-Cu FSSW joint. In joints with no penetration of the upper plate, the joining mainly depends on the metallurgical bonding of the overlapping interface, and the relatively high pressure and welding temperature can produce a stronger joint. Therefore, welding tools with larger diameters have an advantage in the Al-Cu FSSW. Garg et al. [36] studied the effect of tool pin diameter (3.3 mm and 4.95 mm) on shear strength of Al-Cu FSSW joint. The welding tools they used possessed flat shoulders and short pins, as shown in Figure 2b. Their results showed that the joint with the maximum shear strength was fabricated using a pinless tool due to the smaller number of IMCs. Meanwhile, for tools with short pins, the joint strength increased with the increase of pin diameter. The FSSW joint of Al 1050 and pure copper was studied by Ozdemir et al. [38] using a changeable-pin welding tool with a 20 mm diameter shoulder; they found that the difference in pin lengths (2.8, 4 and 5 mm) had a great impact on the mechanical properties of the Al-Cu FSSW joints. Among these, the joint made using a 2.85 mm pin length showed poor mechanical properties. However, in the joint produced by longer pins with lengths of 4 and 5 mm, the extrusion of Cu into the Al plate diffused fully in the joint, while a more uniform Cu accumulation was formed in the keyhole region of the Al side, which resulted in an increase of the joint strength.

In addition to the typical Al-Cu FSSW method, a combined welding tool with a threaded taper interchangeable pin was investigated by Boucherit et al. [45] in an Al-Cu FSSW joint with a Zinc interlayer. Using the lap joint configuration of Cu plate on top of Al plate, they studied the influence of the welding tools with different pin lengths (Figure 2c) on the mechanical properties of the joints. Sufficient pin length increased the effective bonding area of the interface, which was beneficial to improving the mechanical properties of the joint.

It can be concluded that, in the Al-Cu FSSW without penetrating the upper plate, the joint strength is positively related to the diameter of the welding tool within a certain range, while in the joint with penetrating the upper plate, the larger ratio *l/d* of the length of the pin to the diameter makes it easier to obtain higher joint strength.

## 3. Macroscopic Characteristics of Welded Joints

Observing the macroscopic characteristics of joints is the most direct way to analyze the joint formation and evaluate the quality of the joints. In the Al-Cu FSSW process, the materials of the plates, the lap configuration of the FSSW joint (including Al-Cu and Cu-Al), the features of the welding tool, and the welding parameters will influence the surface appearance and cross-section of the joint.

**Figure 3:**
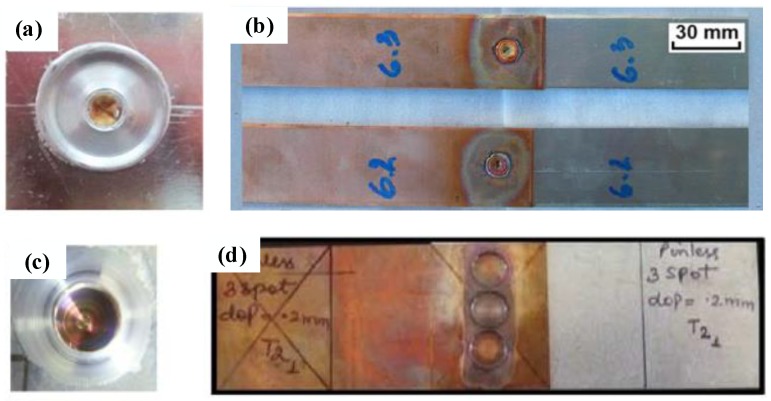
Appearances of Al-Cu FSSW joints with configuration of: (**a**,**c**) Al on the top and Cu on the bottom [42,53]; (**b**) Cu on the top and Al on the bottom [55]; and (**d**) friction stir multi-spot welding [43].

In the FSSW process of Al-Cu dissimilar materials, sufficient heat production and adequate material flow can form a good joint surface, as shown in Figure 3a [42]. The surface of the FSSW joint formed by the cylindrical pin at 2250 rpm shows smooth and shiny features, which is the typical Al-Cu FSSW joint characteristics. In addition, in the investigation conducted by Colmenero et al. [55], when the Cu plate was located in the upper place, due to the higher melting point of Cu, a relatively high temperature was thus required for good plastic flow of the Cu material, which led to the oxidation on the joint upper surface (Cu plate), as shown in Figure 3b. To enhance the tensile load of the joint, multi-point Cu-Al FSSW was studied by Garg et al. [43], and the surface topography of the joint was similar to that of a single solder joint, as shown in Figure 3c.

The cross-section of the FSSW joint can generally be divided into four areas: stir zone (SZ), thermo-mechanically affected zone (TMAZ), heat affected zone (HAZ), and base material (BM). In the center of the joint, the SZ region surrounds the keyhole. Most parts of the TMAZ are at the bottom, and the area around SZ and TMAZ is HAZ, while the BM region is located in the place outside the HAZ away from the weld and occupies the majority part of the joint cross-section [58,59].

**Figure 4:**
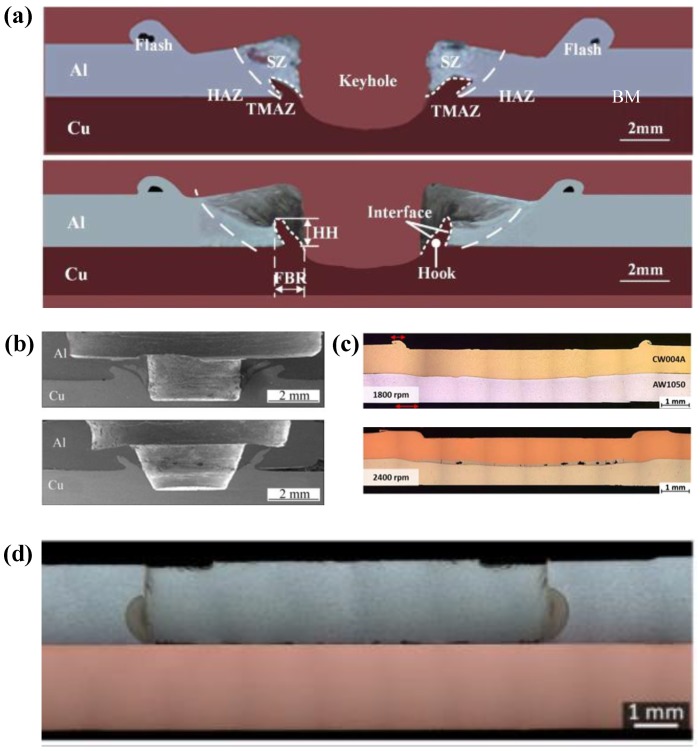
Cross-sections of Al-Cu FSSW joints with (**a**) typical formation with different regions and geometric parameters of the Hook [10], (**b**) joint cross-sections produced by different tool geometries [35], (**c**) joints made by pinless tool with configuration of Cu on the top and Al on the bottom [56], and (**d**) joint of Al-Cu refill-FSSW [57].

In the FSSW of the Al-Cu dissimilar materials, as the welding tool rotates, the lower plate material rises and squeezes into the upper plate to form a Hook structure (Figure 4a). In the FSSW of Al-Cu, the Cu Hook inserted into the upper Al plate is also referred to as the Cu ring by Heideman et al. [37]. The presence of these Cu rings can enhance the interlocking of the two sheets and increase the tensile load of the joint. Zhou et al. [10] conducted a study on Hook in Al-Cu FSSW and described the Hook geometry; they defined the height of the Hook rising into the upper Al plate as the Hook height (*HH)*. As the Hook structure became curled under the squeezing of the material flow, the Hook extended from the edge of the keyhole toward the back of the keyhole, and they defined this length as the fully bonded region (*FBR)*. The ratio of *HH* to *FBR* can be understood as an effective plunging behavior, and their research showed that the ratio was positively correlated with the tensile strength of the joint. The cross-section is also affected by the profile of different welding tools. Results presented by Mubiayi et al. [35] showed that, at the same speed, the Cu ring was deeper in the Al plate under the cylindrical-pin compared to that using the tapered-pin method (Figure 4b). Therefore, the joint obtained by the cylindrical pin exhibited a higher tensile strength. In the Al-Cu dissimilar materials FSSW process, the weld undergoes higher heat input in the configuration of ‘Cu over Al’, providing other parameters remain the same. According to the research of Regensburg et al. [56], the presence of a liquid interlayer was observed, and subsequent re-coagulation during the cooling stage of the joint formed a different topographical feature at the interface than conventional FSSW, as shown in Figure 4c. Compared with common FSSW joints, a layer of liquid metal up to 300 µm thick appeared at the Cu-Al interface, thereby resulting in good wettability and increased contact area of the Cu to Al, bringing about a positive impact on shear strength.

As an important branch of Al-Cu FSSW technology, Al-Cu refill-FSSW has been carried out by some researchers. Due to the use of specially designed welding tools, the materials in the joint undergo the extrusion and refilling stages; thus, the refill-FSSW joint has no keyhole. Cardillo et al. [57] found that the cross-section of the joint showed a different characteristic other than that of the conventional FSSW. The keyhole in the joint was occupied by the refill material. Meanwhile, no Hook was introduced into the cross-section (Figure 4d).

## 4. Microstructure

The type and number of crystal lattices, the radius of the atoms, and the difference in the outer electronic structure affect the metallurgical compatibility of the dissimilar materials [60,61]. The metallurgical compatibility of the two materials in welding depends not only on the mutual solubility of the two materials in liquid and solid state, but also whether the two materials will produce new phase structures or IMCs during the welding process [62,63]. For the Al-Cu dissimilar materials FSSW, the metallurgical compatibility of the two materials is poor, and in addition to the diffusion reaction in the interface of the joint, a large number of IMCs are commonly generated [44,48].

### 4.1. Material Flow

The material flow in FSSW joints has been revealed in several studies through the material tracing method [64,65]. In general, during the FSSW process, the material under the tool shoulder moves downwards, following the rotation of the welding tool; as the material reaches the tip of the tool pin, its flow direction is hindered and then turns outside the pin and upward to form a swirl path due to the constraint of the surrounding hard material. Afterwards, with the upward movement, the flowing material is also decelerated by the obstruction of the pressed material under the rotational shoulder and recirculated along the pin, thereby forming the flow morphology of SZ [66].

**Figure 5:**
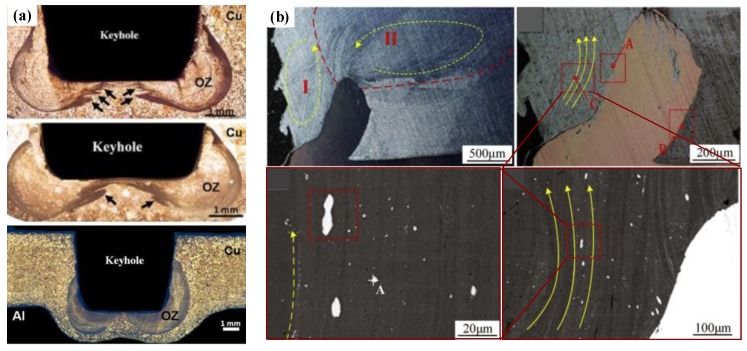
Material flows in (**a**) onion zone (OZ) and typical tubular cups pattern in the Cu-Al FSSW joint [45], (**b**) different regions divided by Cu Hook and their magnifications [11].

When the Cu-Al FSSW is performed, since the below Al material is soft and has high ductility, hard Cu material compresses the Al material below, causing concavity in the lower Al plate in the joint. Therefore, even if the pin length slightly exceeds the thickness of the upper plate, the Cu-Al interface cannot be penetrated. Meanwhile, due to the cold die effect of the underlying hard material and the rotational shear effect of the pin, tubular cups pattern will be formed at the edge of the pin tip [67]. Further studies by Boucherit et al. [45] showed that an obvious onion zone (OZ) structure was formed in the rod-shaped cups by stacking layers of material released at the tip of the pin, as shown in Figure 5a. A more detailed study of Al-Cu FSSW was carried out by Zhou et al. [10]. The joint material flow in their study is shown in Figure 5b. The joint SZ was divided into two regions with counterclockwise material flow by the Cu Hook extruded into the Al plate, and the streamlines of the plasticized metal were clearly observed. In their further study [10], due to the enhanced mechanical stirring effect, most of the IMC particles that entered the SZ with more severe material flow were more refined in the joint obtained under a longer dwell time. In addition, the softening of Cu produced by the large heat input caused the large-sized Cu block to be separated from its matrix and evolved into the multi-phase layered structures through the Al-Cu interaction [68,69].

### 4.2. Interfacial Microstructure Features

**Figure 6:**
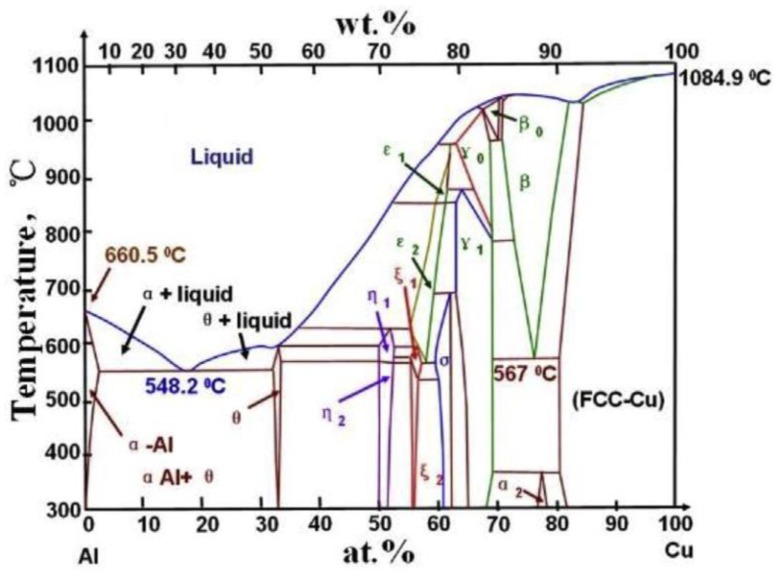
Phase diagram of Al-Cu binary system [11].

According to previous related studies, combined with the Al-Cu binary phase diagram shown in Figure 6 [11], there are six possible equilibrium phases and some metastable intermetallic phases below 500 °C under the corresponding welding conditions [70]. Among these phases, some typical stable phases (Al_2_Cu, AlCu and Al_4_Cu_9_) are more common in solid-phase welding [6,71,72].

In the FSSW of Al-Cu dissimilar materials, the welding thermal cycle and the severe material plastic flow experienced at the interface of the joint lead to different degrees of dissolution and diffusion of Al and Cu, which correspondingly affect the formation and evolution of IMCs at the interface. Due to the dynamic characteristics of FSSW, the formation of IMC at the joint interface is non-uniform and unstable. Furthermore, during the forming process of IMC, a small amount of formed IMC would be stripped off and dispersed in the matrix with the pressure of the interface and the shearing force of the material flow, forming a discontinuous mixed pattern of the IMC layer, especially in FSSW with low heat input. Under the conditions of certain process parameters, a uniform IMC layer with considerable thickness could be constantly formed along the interface [11]. Some studies have discussed the types and thicknesses of IMC at the interface, as shown in Table 3.

In the FSSW of Al-Cu dissimilar materials, the linear velocity of the rotating tool to the joint surface is different, resulting in different heat generation, pressure and material flow at the Al-Cu interface, which correspondingly affects the interfacial IMC features. This effect was discussed by Garg et al. [43] with respect to the FSSW on pure Cu and AA6061 Al alloy, finding that the IMC layer thickness at the interface underneath the shoulder edge was up to 147.7 µm, while the layer was only 6.56 µm thick at the interface of the weld center, according to their previous study [36], the components of the IMC layer were Cu, Al and notable proportion of oxygen. Boucherit et al. [45] obtained a 3.25-µm-thick IMC layer with continuous morphology. The X-ray diffractometry and energy dispersive spectroscopy (EDS) analyses indicated that the IMC layer consisted of Al_2_Cu (2.30 microns) and Al_4_Cu_9_ (0.95 microns) sublayers, which were located on the Al side and the Cu side, respectively.

**Figure 7:**
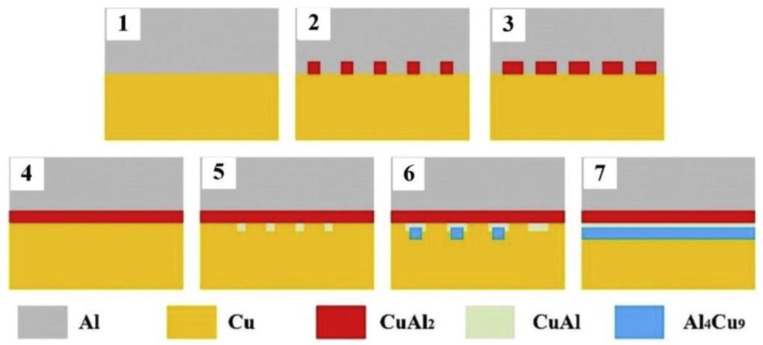
Schematic diagram of IMC evolution at the Al-Cu Hook interface [11].

In the FSSW process, a higher rotational speed corresponds to a higher welding heat input, which also stimulates the growth of IMC at the Al-Cu interface due to the thermal-activated nature of IMC [73]. Zhou et al. [11] studied the effect of rotational speed on the IMC features of the interface in the Al-Cu FSSW joint, and the components of IMCs were identified in follow-up SAED analysis. On the Al-Cu interface back at the keyhole in the joint, a continuous Al_2_Cu-AlCu-Al_4_Cu_9_ layer with thickness of 1.8 µm was obtained at high rotational speed of 3000 rpm, while a discontinuous Al_2_Cu layer with a thickness of 0.2 µm was formed at the low rotational speed of 1500 rpm. They also proposed a prediction model (Figure 7) for the formation sequence of IMCs based on the thermodynamic principle, which was verified by the TEM analysis results of IMC layer samples. They illustrated that discontinuous Al_2_Cu was produced as the initial stage of IMC evolution with the insufficient heat; with the increase of temperature, AlCu nucleated on the surface of Al_2_Cu layer and gradually grew into layers; then the Al_4_Cu_9_ nucleated and develop on AlCu layer; and finally, the continuous Al_2_Cu-AlCu-Al_4_Cu_9_ composite layer formed at the Al-Cu interface in the joint [10,11].

### 4.3. Microstructure of the Al Side

The microstructures of Al-side materials are mainly composed of Al matrix and extruded Cu and Al-Cu IMC particles. The presence of these mixtures makes the microstructures of the Al side complex and irregular.

**Figure 8:**
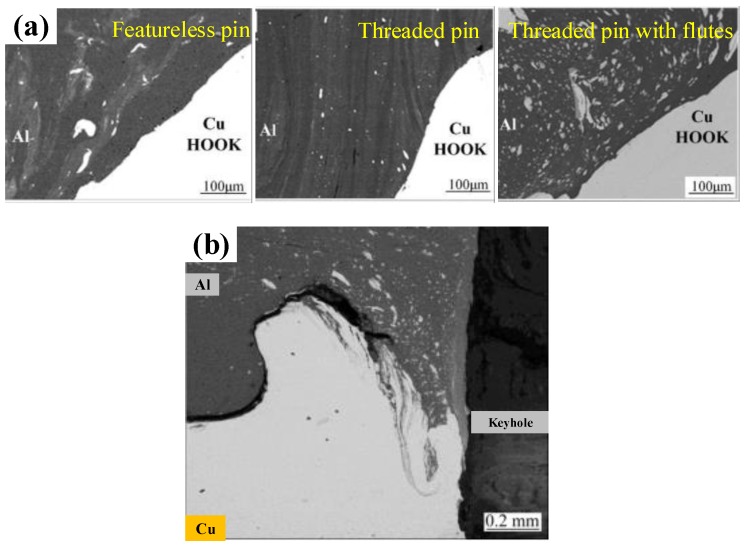
Microstructures of the Al side (**a**) under tools with different pin profiles [42], and (**b**) microstructure of the Al side near the keyhole [35].

The material flow pattern caused by the characteristics of the welding tool has a direct impact on the microstructure of the Al side. In the study of Zhou et al. [42], Cu particles were dragged down from their matrix by the rotating pin and mixed into the Al-side material with the materials flow, as shown in Figure 8a. Compared with the case of the featureless pin, the Cu particles on the Al side of the joint obtained by the threaded pin with three flutes were more evenly distributed due to the more rigorous material flow. Since the threaded pin with flutes possessed the strongest shear force, a large number of large-sized Cu blocks were dragged off from the edge of the Cu sheet, and the existence of flutes widened the flow range of the Cu particles. In addition, at high rotational speeds, the softening of Al and Cu materials caused by high heat generation is more serious, and the material flow is more severe, which also makes it easier for large Cu bulks to separate from the Cu matrix and disperse into the Al side (see Figure 3 in ref. [47]).

In a study carried out by Mubiayi et al. [41], the EDS analysis (see Figure 4 in ref. [41]) showed that the distribution of Cu particles and fine fragments with high Al concentration in the Al side was more evenly dispersed under certain process parameters, which promoted the formation of Al-Cu IMCs. Other studies from the same research group have also confirmed the same phenomenon [35]. In addition, in the vicinity of the keyhole, due to the strongest shear effect caused by the rotating tool during FSSW, Cu particles and Al-Cu IMC presented a highly dispersive distribution (Figure 8b).

### 4.4. Microstructure of Cu Side

In the Al-Cu FSSW, due to the softer characteristics of Al compared to Cu, Cu easily penetrates into the interior of the Al plate through the interface, while it is difficult for the Al material to enter the Cu matrix. Therefore, the Cu-side material is relatively complete and smooth, with no obvious change in the microstructure, which leads to there being less research on the microstructure of the Cu side in the published literature. Meanwhile, these studies have mainly focused on the SZ at the Cu side of the joint. Based on limited research results, the microstructure of the Cu side can be preliminarily observed and analyzed.

On the Cu side of the joint, due to elemental diffusion and metallurgical bonding actions, the position of the Cu matrix edge is partially occupied by the IMC layer, and there is no obvious boundary between the Al and Cu sheets after FSSW. In the study by Heideman et al. [37], in the weak Al-Cu FSSW joint, the IMC close to the Cu side exhibited a fragmentary structure (see Figure 7 in ref. [37]) instead of a layered structure. Meanwhile, they also observed a layer-structure phase at the interface between the Cu matrix and the IMC layer, which could not be identified by the electron microprobe analysis (EMPA).

**Figure 9:**
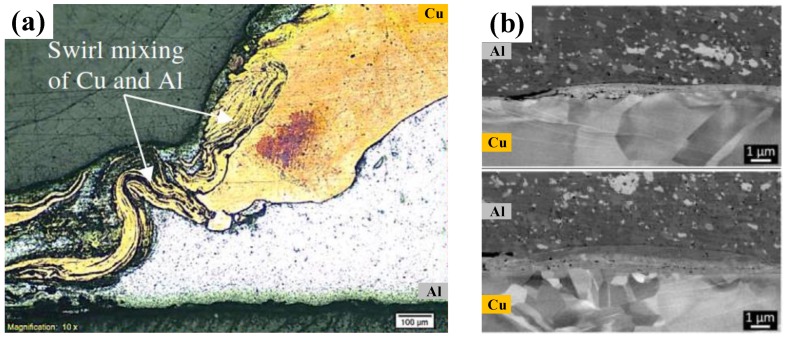
Microstructures of Cu side in (**a**) mixed region [36] and (**b**) Al-Cu interface in refill-FSSW joint [57].

Due to the fierce mechanical action of stirring and mixing in the FSSW and the vertical movement of the upper and lower materials with the pin, a mixed structure of Al-Cu dissimilar materials is commonly formed in the joint under harsh welding conditions. In the Cu-Al FSSW study by Garg et al. [36], the Al material of the lower sheet was rolled up into the Cu sheet and mixed thoroughly to form a swirling layered structure on the Cu side, as shown in Figure 9a. Meanwhile, the dwell time also provided conditions for the diffusion of elements in the mixing zone and the formation of IMCs.

As for the Al-Cu refill-FSSW process, according to the study by Cardillo et al., almost no lower Cu material penetrated through the Al-Cu interface into the Al matrix due to the poor material flow driven by the short sleeve. Therefore, the Cu-side microstructure of the joint was formed with a relatively flat interface, as shown in (Figure 9b).

## 5. Defects in Welds

The welding process method and parameters affect the surface formation of the FSSW joint; the shape and size of the flash, the morphology of the macrostructure, and the interface structure play important roles in the FSSW joint. Up until now, studies on defects in Al-Cu FSSW joints have been rather limited, and have mainly focused on joint surface morphological defects and internal voids.

**Figure 10:**
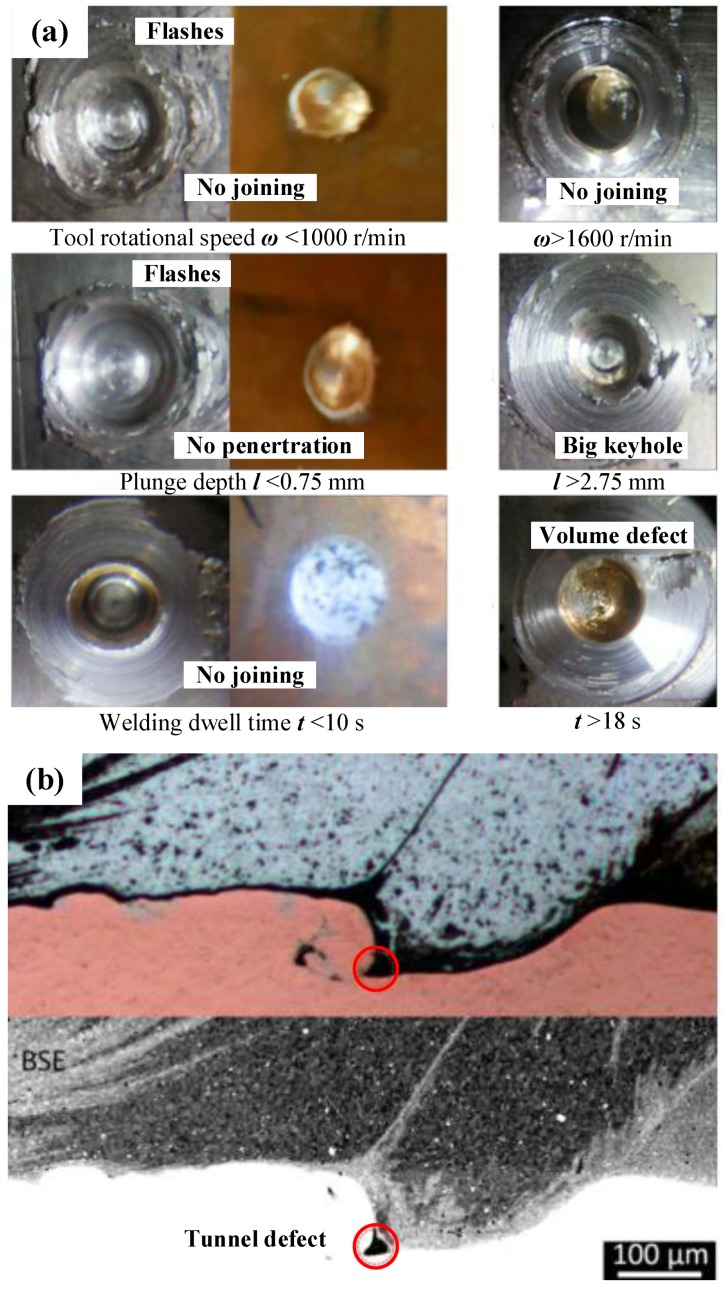
Defects in the (**a**) surface morphology [54] and (**b**) Al-Cu interface of the joint [57].

The main parameters affecting the surface morphology of the FSSW joint are rotational speed, plunge depth and dwell time [74,75]; each of these factors will greatly affect the formation of the joint. In the case of Al-Cu FSSW, the influence of process parameters on the surface morphology of the joint was systematically studied by Siddharth et al. [52,53,54]. In their study, as shown in Figure 10a, the insufficient heat input due to low rotational speed, small plunge depth or short dwell time caused the Al-Cu FSSW joints to be poorly formed, with irregular flashes and rough surfaces, and even the effective joining of the material could not be realized. On the contrary, under excessive heat input process parameters, although the joint connection could be realized, volume defect occurred in the joint surface because of excessive material overflow. In particular, over-penetrating the sheet changed the internal structure of the FSSW joint and increased the volume of the keyhole.

The research on the internal defects of the Al-Cu FSSW joint is mainly concerned with the structural defects in the joint interface. A related study was conducted by Cardillo et al. [57] with the method of refill-FSSW. They confirmed that there were tunnel defects in the interface of the Al-Cu sheets under high rotational speed and large plunge depth, as shown in Figure 10b. In this regard, they held that excessive heat input produced more eutectic structures at the Al-Cu interface [76], and then the liquefied eutectic reduced the shear stress of the welding tool once the peak temperature exceeded the melting point of the eutectic, thereby reducing the fluidity of the plasticized solid material [77,78], which eventually led to tunnel defects at the joint interface.

## 6. Thermal History During Welding

In the FSSW process of Al-Cu dissimilar materials, the welding temperature has a decisive influence on the plastic flow of materials and the formation and evolution of IMCs [79,80,81]. Recording and analyzing the thermal history is essential for revealing and demonstrating the Al-Cu FSSW process. Thus, relevant studies have been carried out.

**Figure 11:**
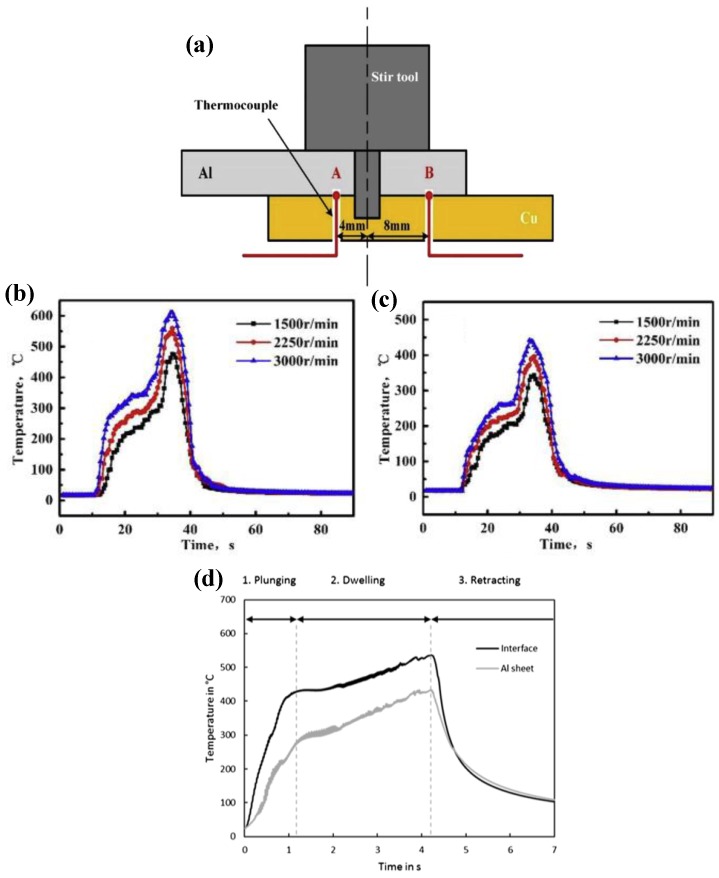
Thermal history measurement (**a**) method and results of (**b**) point A and (**c**) point B of the Al-Cu FSSW joint [11]; (**d**) thermal history of Cu-Al FSSW joint [56].

Zhou et al. determined a higher heat generating welding tool (with a grooved shoulder and a threaded cylindrical pin) in a previous study [42]. Thereafter, they employed two thermocouples to measure the thermal histories of point A (4 mm distance to the FSSW weld center) and point B (8 mm distance to the FSSW weld center), as shown in Figure 11a [11]. They found that the peak temperatures at points A and B both increased with the increase of the rotational speed, and at a rotational speed of 3000 rpm, plunge depth of 0.1 mm and dwell time of 1 s, the peak temperatures at points A and B, respectively, reached 610.5 °C and 441.1 °C, as shown in Figure 11b,c. It is worth mentioning that, in their study, during the plunging process of the welding tool, the viscosity of the plasticized metal material decreased after the temperature reached about 400 °C, and the slipping between the pin and the metal caused a decrease in the ratio of temperature increase [82,83]. With the continuous plunging of the welding tool, the contact between shoulder and sheet caused a sharp increase. Similar results were observed in their further studies [10].

The thermal histories of Cu-Al FSSW joints were investigated by Regensburg et al. [56], who adopted K-type thermocouples to measure the temperatures of the Cu-Al interface and the position inside the Al sheet of 1 mm below the Cu-Al interface. Their study results are presented in Figure 11d, showing that the thermal history during the FSSW process underwent three stages: pluming, dwelling, and retracting; and the peak temperature at the interface reached approximately 535 °C, which was about 100 °C higher than that at the Al sheet, but which was still lower than the Al-Cu eutectic temperature [84,85]. They attributed this to the softening of the Al sheet during the plunging of the welding tool and the resulting sinking displacement of the K-type thermocouple at the interface measurement point.

In the Al-Cu refill-FSSW, the plunge depth of the sleeve has no significant effect on the peak temperature at the Al-Cu interface, while the interfacial peak temperature changes greatly under different rotational speeds; these results were confirmed by Cardillo et al. [57]. In their research, at a rotational speed of 1200 rpm, the corresponding peak temperatures at the plunge depths of 1.6, 1.8, and 2 mm were 460, 481, and 478 °C, respectively. However, at a plunge depth of 2 mm, the peak temperature significantly rose to 504 ° C when the rotational speed increased to 2000 rpm, as shown in Table 4.

## 7. Mechanical Properties

Hardness and tensile strength are obvious indexes for evaluating the quality of FSSW joints. In the FSSW process of Al-Cu dissimilar materials, the welding heat input and material flow significantly affect the microstructure, grain morphology, evolution and thickness of IMCs in different regions of the joint, having decisive influences on the mechanical properties of the joint. Therefore, studying the hardness distribution and the tensile strength of Al-Cu FSSW joint can verify analyses of the welding process and joint microstructure formation, and reveal the welding mechanism, so as to design welding processes, optimize welding parameters, and improve the quality of Al-Cu FSSW joints.

### 7.1. Hardness Distribution

The hardness distribution of the Al-Cu FSSW joint can reflect the temperature, deformation and material flow experienced by the matrix materials during the welding process, and it is also influenced by the distribution of Al-Cu IMCs [86,87]. In a typical Al-Cu FSSW joint, the hardness of Al is less than that of Cu in the BM regions, while in the Hook region, the hardness value has a considerable increase due to the insertion of Cu into the Al matrix. The hardness in the SZ near the keyhole area rise sharply due to the presence of hard and brittle IMCs [88,89,90]. In fact, related studies have shown that the hardness of Al_2_Cu and Al_4_Cu_9_ can be as high as 380 and 525 HV, respectively [91,92]. In the lower Cu sheet, the grains in SZ are fragmented during welding due to the mechanical stirring of the pin, and then recrystallize into small equiaxed grains under the high welding temperature [93,94,95], resulting in a higher hardness in the center of the lower Cu sheet than the BM, and this phenomenon is more remarkable with a more severe stirring. In a further study by Ozdemir et al. [38], the peak hardness in SZ of the Al-Cu FSSW joint produced with 4 mm pin penetration depth was higher than that with a pin penetration depth of 5 mm (see Figure 12 in ref. [38]), which was attributed to the easier formation of IMCs (Al_4_Cu_9_, AlCu and Al_2_Cu) in the joint with 4 mm penetration depth.

**Figure 12:**
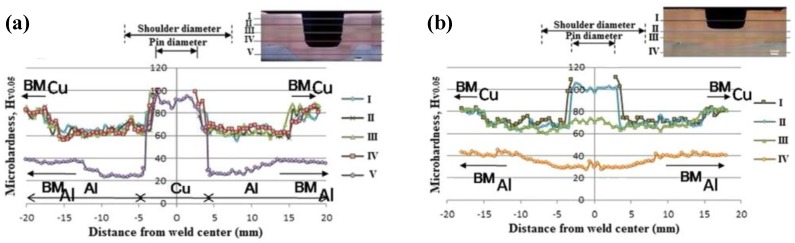
Hardness distributions in Cu-Al FSSW joint with (**a**) 6 mm and (**b**) 2.5 mm pin length [45].

In the Cu-Al FSSW, the hardness distribution of the joint is slightly different from that of the Al-Cu FSSW. As shown in Figure 12, the results obtained by Boucherit et al. [45] illustrated that the different plunge depths have no significant changes in the peak hardness and the hardness distribution of the joints. However, the hardness in the HAZ of the bottom Al sheet is about 10 HV lower than that of the Al BM; this is due to the coarsening of the grain owing to the heating of the material [79]. It is noteworthy that, in the study by Mubiayi et al. [35], due to the Al particles having been dragged into the Cu material, a hardness decrease at the Cu side of SZ was observed. In addition, they also systematically investigated the influence of the probability distribution function (PDF) histogram analysis method on the hardness of different regions of Al-Cu FSSW joints, with the results showing that the profile of the welding tool and the process parameters have a significant effect on the joint hardness distribution [47].

### 7.2. Tensile Properties

Considering the overlap configuration of FSSW joints made of Al-Cu dissimilar materials, the tensile properties of FSSW joints are generally evaluated by shear load. For certain materials of Al and Cu sheets, the geometry of the welding tool and the process parameters are the two most important factors that can significantly affect the tensile properties of the FSSW joints. The majority of the related studies have been carried out on this basis (Table 5), and then achieved improvement of the joint tensile properties by parameter optimization [52,55].

The influences of the tool geometry and the process parameters on the tensile strength of the Al-Cu FSSW joint are mainly achieved by changing the heat generation and material flow of the weld to affect the joint microstructure and the formation and evolution of Al-Cu IMCs. According to the research of Zhou et al. [42], compared with tools with featureless pin and threaded pin with flutes, the tool with threaded pin was helpful for producing larger *HH/FBR* (Figure 4a) at the joint interface, which caused more Cu Hook to be inserted into the upper Al plate and more continuous IMCs to be formed at the Al-Cu interface, thus obtaining a higher shear load of the joint. In the study of FSSW with 3.0 mm thick AA1050 Al and pure Cu sheets conducted by Ozdemir et al. [38], due to the more adequate diffusion from penetrating Cu into Al matrix, a higher tensile load (3950N) of the joint was obtained under the tool with the pin length of 4mm than under that of 5 mm.

**Figure 13:**
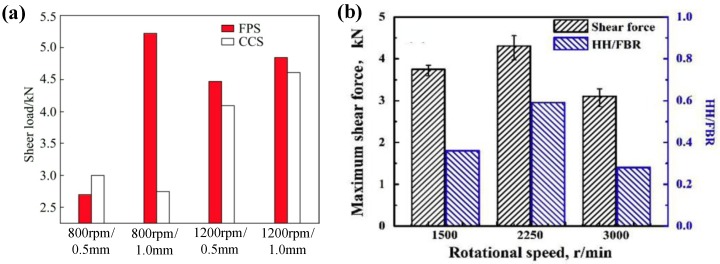
Tensile properties of joints under (**a**) different rotational speeds and shoulder plunge depths [35] and (**b**) different rotational speeds [11].

The effect of process parameters on the tensile properties of Al-Cu FSSW joints were verified by Heideman et al. [37]. They found that the change in rotational speed had the most obvious effect on the shear load of the joint; when the speed was increased from 1000 to 2000 rpm, the improvement of the shear load rose as high as 150%. Additionally, the tensile properties of the Al-Cu FSSW joints are also affected by the variation of the shoulder plunge depth. In the study by Mubiayi et al. (Figure 13a) [35], under welding conditions with certain rotational speeds (800 and 1200 rpm) and welding tools (flat shoulder and flat pin (FPS) and concave shoulder and conical pins (CCS)), the joint shear load increased as the plunge depth increased from 0.5 mm to 1.0 mm, except for welding with the CCS tool at 800 rpm. Zhou et al. [10,11] discussed the effect of rotational speed and dwell time on the tensile properties of Al Cu FSSW joints. In their research, proper rotational speed can result in higher *HH/FBR* ratio, which can enhance the mechanical interlock, and correspondingly improve mechanical properties of Al-Cu FSSW joint. As shown in Figure 13b, with the rotational speed of 2250 rpm, the joint shear load reached a maximum of 4304N [11]. Meanwhile, in the Al-Cu FSSW, appropriate dwell time can improve the shear load of joint by optimizing the pattern of look and improving the continuity of IMCs. When a long dwell time was adopted, the increasing thickness of little IMCs and the curling of the Cu hook lead to the increase of *HH/FBR*, weakening the mechanical interlock between Al and Cu plates, and reducing the joint shear load [10].

## 8. Summary and Outlook

In this paper, the current studies on Al-Cu FSSW (tool features, macroscopic characteristics of welded joints, microstructures, defects in welds and mechanical properties of joints) were reviewed. Many efforts have been made by researchers to achieve tight bonding and higher joint quality of Al-Cu FSSW. Specifically, in order to compromise the differences in physical and chemical properties between Al and Cu dissimilar materials, achieve good metallurgical bonding in FSSW joints, and obtain defect-free joints, systematic studies have been carried out with respect to many aspects. Although some satisfactory results have been reported in the literature, there are still some gaps between the present Al-Cu FSSW technology and its actual industrial application.

As an effective and efficient welding method, FSSW has great potential in industrial applications. The demands of reliable Al-Cu joints also drive the rapid development of the FSSW for joining dissimilar materials. In view of this, based on the published research results, some brief suggestions are put forward for future research with regard to several main aspects, as follows.

(1) Material flow during Al-Cu FSSW

In the Al-Cu FSSW process, the mixing of dissimilar materials and the microstructure formation of joints closely depend on the flow of materials, which are all driven by the rotating tool. At present, the research on material flow in Al-Cu FSSW is still insufficient, and needs to be further studied in order to better understand the welding process.

(2) Al-Cu FSSW thermal history

Welding heat input and the thermal cycle of the welding process are essential for FSSW. Friction and heat generation during the Al-Cu FSSW process have significant and complex impacts on the subsequent material flow and the evolution of interface IMCs. Multi-point temperature measurement and numerical simulation of temperature field are beneficial to understanding the FSSW process of Al-Cu.

(3) Addition of intermediate layer

The differences of physical and chemical properties between Al and Cu dissimilar materials are unavoidable. Thus, adding an intermediate layer in the Al-Cu FSSW process can be a good solution. The mechanical properties of joints can be further improved by adding intermediate layers to inhibit the development of Al-Cu IMCs. Furthermore, strength improvement of the joint interface may be achieved by adding an intermediate layer of high-strength materials.

(4) Auxiliary methods

The FSSW process requires fewer welding environment and operating conditions, in that it does not need to be performed in a specific area and space. Therefore, exploratory studies using auxiliary methods such as auxiliary heating and ultrasonic vibration can be considered.

(5) Functionalization research of Al-Cu FSSW joints

FSSW joints of Al-Cu dissimilar materials are mainly used for meeting functional requirements. The current research on the quality evaluation of joints mainly focuses on the macroscopic morphology, the microstructure of joint, and mechanical properties such as hardness and tensile strength. In the future, it would be worthwhile to systematically conduct electrical conductivity, corrosion resistance, and other functionalization studies of Al-Cu FSSW joints.

## Figures and Tables

**Table 1:** Abbreviations of technical terms presented in this study.

Technical Terms	Abbreviations
Aluminum	Al
Base material	BM
Conical pin and concave shoulder	CCS
Copper	Cu
Electron microprobe analysis	EMPA
Energy dispersive spectroscopy	EDS
Electromagnetic pulse welding	EMPW
Flat pin and flat shoulder	FPS
Friction stir spot welding	FSSW
Friction stir welding	FSW
Fully bonded region	FBR
Heat affected zone	HAZ
Hook height	HH
Hook interface back to the keyhole	IBK
Hook interface facing the keyhole	IFK
Intermetallic compound	IMC
Onion zone	OZ
Probability distribution function	PDF
Stir zone	SZ
Thermo-mechanically affected zone	TMAZ

**Table 2:** Welding tool features used in the Al-Cu FSSW.

Shoulder		Pin			Joint Strength (Shear Force, kN)	Ref.
Diameter (mm)	Morphology	Diameter (mm)	Length (mm)	Morphology		
10	Concave	4	1.83/ 2.60	Threaded	1.7/ 2.0	[37]
20	Flat	5	2.8/ 4.0/ 5.0	Threaded	1.8/ 3.9/ 3.2	[38]
10	Concave	3	4.5		Close to 4.8	[39]
15	Flat/ Concave	5	4	Flat/ Conical	5.2/ 4.8	[35,41,44,46,47]
16	Flat	6	1.2	Cylindrical	2.6	[40,48]
16	Flat	6	1.5/ 2.5/ 6.0	Tapered and threaded	2.8/ 3.4/ 4.6 (with 0.5 mm Zinc layer)	[45]
10	Flat	Pinless tool			1.7 (Shear force)/ 0.3 (Cross tensile force)	[43]
10	Flat	Pinless tool/			1.9 1.5/1.1 1.6/1.3	[36]
		3.3/ 4.95	0.2/0.4 0.2/0.4	Cylindrical/ Cylindrical		
18	Flat	5	4.5	Cylindrical	4.5	[49]
16	Flat	6	1.5	Cylindrical	3.8	[50,51,52,53]
16	Flat	6	1	Cylindrical	3.8	[54]
14	Concave	4.6	2.85	Cylindrical/ threaded pin/ threaded pin with flutes	2.7/ 4.3/ 3.1	[10,11,42]
10	Concave	3	4.5	Cylindrical	4.8	[55]
12	Flat	8	0.3-0.4	Cylindrical	Close to 3.4	[56]
Refill-FSSW 14.5 mm (clamping ring) 9 mm (sleeve)		6		Threaded	7.1	[57]

**Table 3:** Types and thickness of the interfacial IMC in the Al-Cu FSSW joints.

Materials	Interface Position	IMC Types	IMC Thickness	Ref.
Pure Cu/ Pure Al	Cu-Al interface: 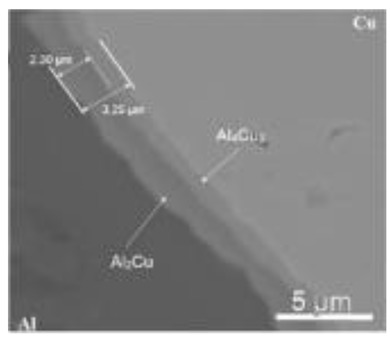	Al_2_Cu Al_4_Cu_9_	Total: 3.25 µm; Al_2_Cu: 2.30 µm; Al_4_Cu_9_: 0.95 µm	[45]
Pure Cu/ AA6061 Al	Cu-Al interface: 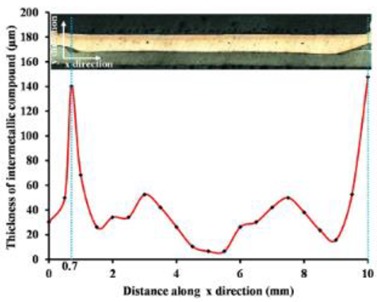	Major elements in IMC layer are Al, Cu and notable amount of oxygen.	Varied from 6.56 to 147.70 µm	[43]
AA1060 Al/ C11000 Cu	Al-Cu Hook interface: 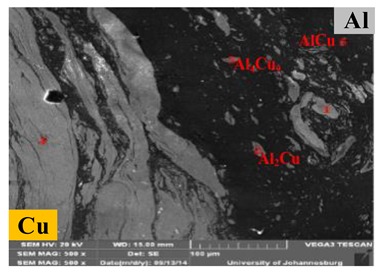	FPS/800 rpm: AlCu_3_, Al_4_Cu_9_, Al_2_Cu, Al_3_Cu_2_, Al_2_Cu_3_, AlCu; FPS/1200 rpm: AlCu_3_, Al_4_Cu_9_, Al_2_Cu, AlCu; CCS/800 rpm: AlCu_3_, Al_4_Cu_9_, Al_2_Cu, Al_3_Cu_2_; CCS/1200 rpm: AlCu_3_, Al_4_Cu_9_, Al_2_Cu, Al_3_Cu_2_	Not mentioned	[35]
CW004 Cu/ AW1050 Al	Cu-Al interface: 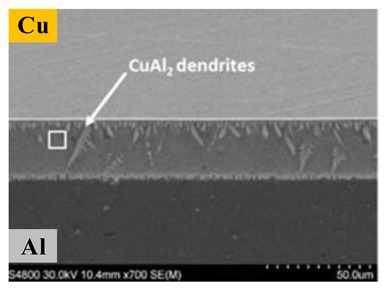	2200 rpm: melt layer (within Al_2_Cu dendrites); 2400 rpm: melt layer (within Al_2_Cu, AlCu and Al_4_Cu_9_)	2200 rpm: melt layer >100 µm; 2400 rpm: melt layer >300 µm; The thickness of IMC inside the melt layer < 5µm	[56]
1060 Al/ T2 Cu	Al-Cu Hook interface: 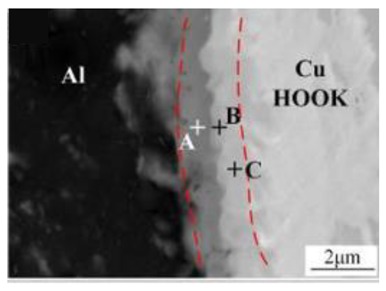	Al_2_Cu AlCu Al_4_Cu_9_	Featureless pin: IFK: 1.3 µm (Al_2_Cu- AlCu-Al_4_Cu_9_); IBK: 0.6 µm (Al_2_Cu); Threaded pin: IFK: 2.8 µm (Al_2_Cu- AlCu-Al_4_Cu_9_); IBK: 1.0 µm (Al_2_Cu- AlCu); Threaded pin with flutes: IFK: 1.9 µm (Al_2_Cu- AlCu-Al_4_Cu_9_); IBK: 1.4 µm (Al_2_Cu- AlCu-Al_4_Cu_9_)	[42]
1060 Al/ T2 Cu	Al-Cu Hook interface: 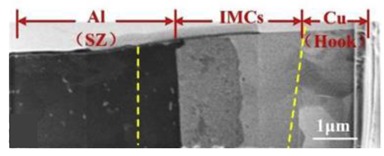	Al_2_Cu AlCu Al_4_Cu_9_	1500 rpm: IFK: 2.0 µm (Al_2_Cu- AlCu-Al_4_Cu_9_); IBK: 0.2 µm (Al_2_Cu); 2250 rpm: IFK: 2.8 µm (Al_2_Cu- AlCu-Al_4_Cu_9_); IBK: 1.0 µm (Al_2_Cu- AlCu); 3000 rpm: IFK: 3.4 µm (Al_2_Cu- AlCu-Al_4_Cu_9_); IBK: 1.8 µm (Al_2_Cu- AlCu-Al_4_Cu_9_)	[11]

FPS—Flat pin and flat shoulder; CCS—Conical pin and concave; IFK—Hook interface facing the keyhole; IBK—Hook interface back to the keyhole.

**Table 4:** The peak temperatures in different parameters studied by Cardillo et al. [57].

Rotational Speed (rpm)	Plunge Depth (mm)	Dwell Time (s)	Peak Temperature at the Al-Cu Interface (°C)
1200	1.6	0	460
1200	1.8	2	481
1200	2	2	478
2000	2	2	504

**Table 5:** Tensile properties of FSSW joints of Al-Cu dissimilar materials.

Materials	Max. Shear Load (N)	Tool Features	Welding Parameters: R/rpm (Rotational Speed), D/mm (Plunge Depth), T/s (Dwell Time)	Ref.
6061-T6 Al (1.5 mm) and pure Cu (1.5 mm)	2080 N	Concave shoulder (10.0 mm diameter) and threaded pin (2.6 mm length)	R = 2000 rpm D = 0.13 mm T = 3 s	[37]
AA1050 Al (3.0 mm) and pure Cu (3.0 mm)	3950 N	Flat shoulder (20.0 mm diameter) and threaded pin (4.0 mm length)	R = 1600 rpm T = 10 s	[38]
AA1060 Al (3.0 mm) and pure Cu (3.0 mm)	5225 N	Flat shoulder (15.0 mm diameter) and flat pin (4.0 mm length)	R = 800 rpm D = 1.0 mm T = 10 s	[35,41]
5083 Al (1.5 mm) and C10100 Cu (1.5 mm)	2600 N	Flat shoulder (16.0 mm diameter) and cylindrical pin (1.2 mm length)	R = 1250 rpm D = 0.7 mm T = 12.5 s	[40]
Pure Cu (5.0 mm) and pure Al (2.0 mm)	4610 N	Flat shoulder (16.0 mm diameter) and tapered pin with thread (4.0 mm length)	R = 1400 rpm T = 8 s	[45]
Pure Cu (0.5 mm) and AA6061-T6 Al (0.5 mm)	1728 N	Pinless tool with flat shoulder (10.0 mm diameter)	R = 2500 rpm D = 0.2 mm T = 4 s	[43]
5083 Al (1.5 mm) and C10100 Cu (1.5 mm)	1120 N	Flat shoulder (16.0 mm diameter) and cylindrical pin (1.2 mm length)	R = 1250 rpm D = 0.9 mm T = 12 s	[48]
5083 Al (1.5 mm) and C10100 Cu (1.5 mm)	3780 N	Flat shoulder (16.0 mm diameter) and cylindrical pin (1.5 mm length)	R = 1000 rpm D = 0.5 mm T = 18 s	[50]
5052 Al (1.5 mm) and C27200 Cu (1.6 mm)	3908 N	Flat shoulder (16.0 mm diameter) and cylindrical pin (1.0 mm length)	R = 1350 rpm D = 0.95 mm T = 13.5 s	[54]
5086 Al (1.5 mm) and C10100 Cu (1.6 mm)	2190 N	Flat shoulder (16.0 mm diameter) and cylindrical pin (1.5 mm length)	R = 1100 rpm D = 0.55 mm T = 11.5 s	[52]
1060 Al (2.0 mm) and T2 Cu (2.0 mm)	4304 N	Concave shoulder (14.0 mm diameter) and cylindrical pin with thread (2.85 mm length)	R = 2250 rpm D = 0.1 mm T = 5 s	[10,11,42]
AA5083 Al (2.0 mm) and Cu DHP (2.0 mm)	7110 N (Refill-FSSW)	Threaded tool with clamping ring (14.5 mm diameter), sleeve (9.0 mm diameter) and pin (6.0 mm diameter)	R = 1200 rpm D = 2 mm (Sleeve plunge depth) T = 2 s	[57]
Pure Cu (3.0 mm) and AA1050-H24 (3.0 mm)	4830 N	Concave shoulder (10.0 mm diameter) and cylindrical pin (4.5 mm length)	R = 1255 rpm D = 0.2 mm T = 4 s	[55]
